# The Potential of ICP-MS as a Complementary Tool in Nanoparticle–Protein Corona Analysis

**DOI:** 10.3390/nano13061132

**Published:** 2023-03-22

**Authors:** Ana Fuentes-Cervantes, Julia Ruiz Allica, Francisco Calderón Celis, José M. Costa-Fernández, Jorge Ruiz Encinar

**Affiliations:** Department of Physical and Analytical Chemistry, University of Oviedo, Avda. Julián Clavería 8, 33006 Oviedo, Spain

**Keywords:** protein corona, ICP-MS, nanomaterials, proteins, nanoparticles

## Abstract

The prolific applicability of nanomaterials has made them a common citizen in biological systems, where they interact with proteins forming a biological corona complex. These complexes drive the interaction of nanomaterials with and within the cells, bringing forward numerous potential applications in nanobiomedicine, but also arising toxicological issues and concerns. Proper characterization of the protein corona complex is a great challenge typically handled with the combination of several techniques. Surprisingly, despite inductively coupled plasma mass spectrometry (ICP-MS) being a powerful quantitative technique whose application in nanomaterials characterization and quantification has been consolidated in the last decade, its application to nanoparticle–protein corona studies is scarce. Furthermore, in the last decades, ICP-MS has experienced a turning point in its capabilities for protein quantification through sulfur detection, hence becoming a generic quantitative detector. In this regard, we would like to introduce the potential of ICP-MS in the nanoparticle protein corona complex characterization and quantification complementary to current methods and protocols.

## 1. The Context of the Protein Corona

### 1.1. The Protein Corona Formation

Nanotechnology has experienced enormous growth in recent decades, bringing forward the surging use of engineered nanoparticles as advantageous novel materials in numerous fields such as biomedicine, cosmetics, pharmacology, food, or agriculture [1,2]. Its unrestrained used has resulted in the ubiquity of these nanomaterials in the environment [3] and biological systems [4], and thus nanoparticles may potentially be inhaled, ingested, or taken up through the skin into the body [5]. Then, when nanoparticles come in contact with biological fluids, they become covered by a complex layer of biomolecules, such as proteins, lipids, or sugars, forming a sort of “bio-corona” [6,7]. Within the biological corona complexes formed in biological systems, the predominant and most studied molecules are proteins, which on their own form the so-called nanoparticle protein corona complex (NPPC) [5,8]. The protein corona defines the biological identity of the nanoparticle, the NPPC being the entity that finally interacts with and is “seen” or recognized by the cell [6].

**Figure 1:**
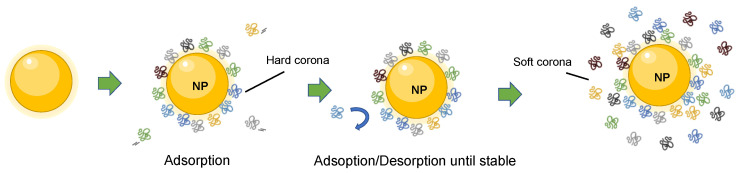
Scheme representation of the dynamic formation of nanoparticle–protein corona complex. Proteins in the inner layer have high affinity for the nanoparticle surface and form the hard corona. The proteins in the outer layer form the soft corona, which shows dynamic changes due to exchanges with free proteins in the environment.

Upon introduction in a biological fluid, the process of protein adsorption is an almost instantaneous event given the higher binding energy of the nanoparticle surface compared to the surrounding biological environment (Gibbs free energy drives the complex stability) [9,10]. The formation of the corona, driven by non-covalent forces, is dynamic and changes over time because of continuous association and dissociation processes given the different affinity of the proteins. Initially, higher concentration proteins will interact with the nanoparticle, but will eventually be displaced by higher-affinity proteins until reaching an equilibrium, when protein exchanges would not affect the composition of the corona [6,9,11,12]. In fact, although the formation of the corona takes place over the period of an hour, occurring minor changes for around 12 h [13], plasma proteins have been found in the corona already within the first minute of exposure [14]. Interestingly, the composition of the corona at such early stages does not significantly change over time in protein identities, but in quantities [14]. The dynamic nature of these phenomena can be described by using the “hard” and “soft” corona concepts. Those proteins with high affinity that form the closest layer to the nanoparticle surface are called the hard corona. This constitutes a tight, strong, nanoparticle–protein binding that is highly stable of very rapid formation. Low-affinity proteins forming an external layer, which are not bound to the nanoparticle surface but have a certain degree of interactions, are called the soft corona [4,6,9] (Figure 1). It takes more time to constitute, and it is more unstable and highly dynamic, being more complex in its study.

Nanomaterial interactions with proteins can be controlled by different strategies, nanoparticle surface functionalization being the most common one [15]. This is because protein corona formation is a process commonly considered problematic, and thus great effort is being invested by the scientific community on designing and engineering coatings that minimize protein adsorption on nanomaterial surfaces as much as possible [16].

### 1.2. The Biological Impact of the Protein Corona

The interactions between nanoparticles and proteins have significant biological consequences to the original nanoparticle and to the native proteic environment. On the one hand, the formation of the protein corona on the nanoparticle surface changes the nanoparticle physicochemical properties. It changes the size, shape, and even the aggregation state of the nanoparticle, which in turn affects how proteins are oriented and presented to the biological targets [17]. On the other hand, interactions with nanoparticles may trigger protein aggregation or conformational changes, which may impact the protein functions and their interaction with other biomolecules, and may even result in an immune response to eliminate the circulating nanoparticles. In this regard, changes in protein stability and enzyme activity have been observed when immobilized on the nanoparticle surface [6].

Upon entrance in the biological media, nanoparticles are involved in a myriad of biological processes. The formation of the protein corona creates a different structure that will drive and influence the behavior and interaction of the nanoparticles with biological systems. Thus, the protein corona can alter the interactions of the nanoparticle with the cell surface moieties (e.g., antibodies), promoting or inhibiting the uptake of the nanoparticle by the cell [18]. For instance, in a case of study with silica nanoparticle, it was observed that the formation of the protein corona reduced the cell uptake by weakening their cell membrane adhesion [19]. The protein corona also plays an important role in targeting the nanoparticles to different organs [13].

The biological impact of the nanoparticle interactions with proteins is particularly relevant in the development and use of the NPPC in biomedicine. The small size of nanoparticles gives them the ability to efficiently access cell compartments, which has important biomedical implications, leading to important developments in the field of nanomedicine [20,21]. The feature of the protein corona, which can direct the nanoparticles to specific organs or locations, can be used to design NPPC that can be placed anywhere desired in the organism or in the cell. This final location will then determine the effect of the NPPC and the pathways or processes it gets involved in, disrupts, or promotes. Likewise, it can be used as a vehicle for drug delivery [18], or for the promoted accumulation of nanoparticles in specific areas, e.g., tumors, and serve as a biomarker or therapeutical target [9]. Naturally, the presence of nanoparticles in biological systems and their use in biomedicine brings a potential safety and toxicological risk. Even for nanoparticles without the protein corona, cellular uptake is related to cytotoxicity, being affected by properties like colloidal stability or nanoparticle surface charge. In fact, positively charged nanoparticles tend to have higher cellular uptake as well as a higher cytotoxicity than negatively charged nanoparticles [22].

## 2. Traditional Approaches to Study and Characterize the Nanoparticle–Protein Corona Complex

Protein–nanoparticle interactions are highly determined, not only by the environment composition and dynamics, but also by the nature and structure of the nanoparticles. Therefore, characterization of the starting nanoparticle is a key issue that must be investigated and understood before affording the study of the protein corona.

### 2.1. Nanoparticle Characterization

Nanoparticles’ physicochemical properties control the formation and composition of the protein corona. Whereas the extent of the impact of each property on the protein corona composition and disposition is not completely understood, there is an extended consensus that no nanoparticle property alone drives the formation of the corona, but all of them together [23]. Nanoparticle protein adsorptions are mainly driven by non-covalent forces, affinity constants of proteins and protein structure thermodynamics [24]. Thus, the nanoparticle surface constitutes a major factor influencing the protein corona [25]. In contrast to the previously commented direct influence of nanoparticle surface charge in the toxicity and biodistribution of nanoparticles, this parameter does not seem to be so influential on the formation and eventual biological effect of NPPC [22,25].

The hydrophobicity of a nanoparticle’s surface is, on the contrary, a significant factor limiting the amount and identity of the proteins bound to the nanoparticle. In this regard, for instance, Lynch et al. found that when plasma is incubated with low hydrophobic copolymer particles, virtually no protein is retrieved, while significant amounts of protein are consistently bound to hydrophobic particles [26]. Nanoparticle surface chemistry is also a driving parameter in the formation of the characteristics of the protein corona, as it controls the number of possible binding sites [12]. Nanoparticles commonly need to be functionalized with ligands and biomolecules that affect their solubility, stability, and define and control their biocompatibility and biological interactions [27]. This functionalization eventually affects the formation of the protein corona (because it largely confers a stealth character on the nanoparticle, protecting it and making it inaccessible to proteins) and could be crucial when developing nanoparticles with biological or therapeutical applications [28]. Walkey et al. observed that differently charged ligands have more adsorption capabilities than neutral ligands [17], whereas Galmarini et al. observed a higher number of proteins forming the corona when nanoparticles were initially functionalized with citrates rather than with DMSA, stating the influence of the nanoparticle functionalization on the corona protein composition [25]. Despite the clear relevance of the adequate characterization of nanoparticle functionalization, there are yet no standard methods available to determine nanoparticle/surface biomolecule ratios [29]. The surface charge of nanoparticles also seems to be a key parameter affecting nanoparticle–protein interactions. Indeed, Lundquist et al. studied the PC composition formed in three different polystyrene particles: with positive (amine-modified), neutral (the plain unmodified particles), or negative (carboxyl-modified) charges. Remarkably, they found a certain dependence of the resultant protein corona depending on surface charge and zeta potential, and they were able to identify adsorption patterns and similarities [30].

Interestingly, several works have found that nanoparticle size seems to be more relevant than their functionalization in the formation of the protein corona. Tenzer et al., for instance, determined nanoparticle size to be dominant in the formation of the protein corona over surface functionalization, which in turn was dominant over exposure time [14]. When comparing nanoparticles of different sizes with the same functionalization, Walkey et al. observed that the steric hindrances formed in larger nanoparticles hindered the adsorption of proteins, which was more favored in smaller ones [17]. Nanoparticle shape is a key parameter that has, for some reasons, been disregarded in protein corona investigations. However, different studies evidenced that protein corona formation is also dependent on nanoparticle morphology. As an example, Deng et al. found that protein binding rate is different for TiO_2_, SiO_2_, and Zn nanoparticles; TiO_2_ nanorods; and TiO_2_ nanotubes, despite these nanoparticles having similar surface charges [31].

**Figure 2:**
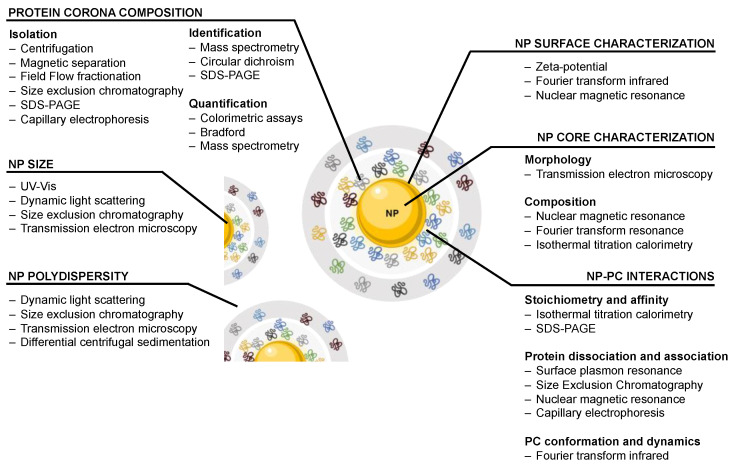
Common methods used to characterize nanoparticle–protein corona complexes.

In this regard, common approaches to study and determine nanoparticle size and size distribution include dynamic light scattering (DLS) and differential centrifugal sedimentation (DCS) (Figure 2). DLS is a fast and general-purpose method of nanoparticle dispersions, and it is of low resolution, though it is limited in terms of sample concentration, small nm shifts in size, non-spherical particles, or the dynamic range of nanoparticle size [32]. In the case of most precise nanoparticle size studies, more accurate techniques such as DCS are required.

In contrast to DLS, DCS allows the resolution of multiple peaks in polydisperse samples as well as the possibility to carry out the analysis even in the presence of large biomolecules, as in the case of protein corona studies [23]. Transmission electron microscopy (TEM) is routinely used to determine nanoparticle size, shape, and size distribution. In fact, it can also provide information on nanoparticle aggregation, as well as morphology. This is particularly relevant because, as observed by Nandakumar et al. when they studied the protein corona formation on model AuNPs, the AuNP morphology conditioned the identity of the proteins forming the protein corona based on their size and structure [33]. Interestingly, different morphologies resulted in differences in both the identities and the quantitates of the proteins forming the corona. In brief, it is therefore fundamental to control nanoparticle size, shape, and surface properties to control nanoparticle behavior and produce NPPCs in a systematic and reproducible way (see Figure 2).

### 2.2. Protein Corona Composition

When nanoparticles enter biological media, the number of proteins they may get in contact with is in the thousands. However, they cannot all fit onto the nanoparticle surface: typically, less than 100 proteins form the hard corona in nanoparticles of >10 nm [34,35], resulting in the possibility of subpopulations of a certain nanoparticle with different protein coronas, potentially showing different in vivo behavior [11]. The impact of the environment in the protein corona composition is not limited to the upfront formation of the corona, but when the NPPC changes its location, the composition of the corona is affected by the new environment, leading to changes in the identity and quantity of the proteins that formed the hard corona. Protein composition variation is even more pronounced in the soft corona, as they have lower and more variable resident times. Therefore, characterization and understanding the size and composition of the protein corona is critical to predicting the behavior of the NPPC in biological systems, and to design nanoparticles with specific properties, such as improved biocompatibility or targeted drug delivery. In this regard, the identification of both major and minor proteins that form the corona is essential, as is the study of their abundance and their affinity, e.g., to study their competition to bind when the system is under kinetic or thermodynamic influence, or understanding the NPPC fate in biological systems or role in biomedicine [8,18].

It should be stressed that identification of the different proteins is not enough to obtain a comprehensive characterization of the protein corona. The dynamism in the protein corona composition (particularly the soft corona), as well as the reasonable assumption that the most abundant proteins will define the biological roles of the NPPC, demands quantitative data in order to achieve a comprehensive characterization. Additionally, considering that protein corona composition is driven by the affinity and interactions between nanoparticle and proteins, there is not necessarily a correlation in protein abundance in the media and in the corona [17,18]. Because of these challenges that entail the study of NPPCs in biological systems, unbound proteins present in the media must be removed prior to the proteomic study of the corona, without affecting or altering the NPPC composition. The most common methods used to isolate protein corona-coated nanoparticles include centrifugation, size exclusion chromatography, magnetic separation, and field-flow fractionation [36]. In this regard, purification and isolation methods such as centrifugation or chromatography are usually preferred to separate proteins from complex mixtures and plasma. It must be noted though that these purification processes may also result in the loss of the weakly bound proteins forming the soft corona [37].

Quantification of proteins forming the corona can be performed through standard approaches such as colorimetric assays, such as BCA, Bradford or Lowry [38]. These techniques are based on the complexation of the protein with metal ions or dyes (respectively), leading to a color change calibrated with a reference standard protein such as BSA. These approaches provide information regarding the total protein amount present in the samples but are limited because of potential reactivity differences between calibrant and the assayed protein, leading to errors in protein estimation. Gel electrophoresis has also been widely employed for the separation of proteins of the corona from the nanoparticles, and their identification and classification by molecular weight. Alternatively, Benetti et al. assayed different detachment approaches, such as using SDS or isopropyl alcohol and NaOH, but none of them were able to fully detach the complete amount of proteins from the nanoparticle surface [39]. Thus, there are some parts of the corona that will not be revealed. This is why the predominant approach to carrying out the qualitative and quantitative analysis of the protein corona is mass spectrometry (MS), as proteins are directly digested in the NPPC, hence it is more efficient than the detachment approaches required for Lowry or SDS-PAGE assays.

**Figure 3:**
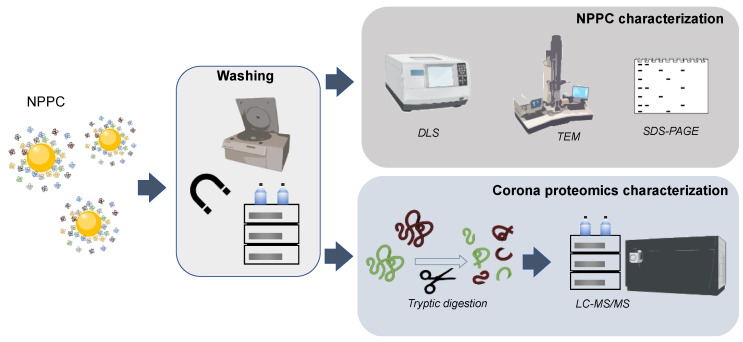
Schematic representation of a typical workflow for the synthesis and characterization of the NPPC.

In a typical workflow (Figure 3), before the LC-MS protein corona characterization, NPPC samples are first washed before their characterization by DLS and TEM to investigate the homogeneity/heterogeneity of the PC-coated nanoparticles, and SDS-PAGE to study the protein corona profiles (after separating the proteins from the nanoparticle) [18]. Next, LC-MS/MS is used to characterize the composition of the protein corona (identification of the different proteins adsorbed onto the nanoparticle) employing proteome approaches. This study requires the enzymatic digestion of the proteins, either in solution directly to the NPPC, or in-gel after the SDS-PAGE. Before protein corona characterization by MS approaches, isolation of the PC-coated nanoparticles must be carried out by size exclusion chromatography, magnetic separations, field flow fractionation, or centrifugation, the latter being the most widely used approach. Then, the NPPC is digested and analyzed with LC-MS. Identification is carried out through sequencing of the proteins following common proteomics protocols [7,40]. MS can also provide quantitative protein information, though regular LC-ESI-MS/MS workflows provide only semiquantitative data by label-free methods, which estimate protein abundances from the peptide counts and ion intensities in a single LC-MS/MS analysis. Absolute quantities of the proteins forming the corona would require specific standardization, which is constricted when addressing the analysis of a high number of proteins, as is the case in protein corona studies [41].

### 2.3. Nanoparticle–Protein Corona Interactions

The biological relevance of protein–nanoparticle interaction in the formation of the protein corona and the relevance and need for a proper characterization of such interactions, which are yet not completely understood, has already been remarked [40]. These interactions are affected by diverse factors such as protein identities, quantities, affinities and specificities, exchange rates, and redistribution, all these determining the eventual biological effect of the NPPC. Study and characterization of the protein corona formation and interaction with the nanoparticle is usually carried out through indirect approaches, which are based on comparing the nanoparticle before and after the formation of the protein corona. Then, changes in the nanoparticle properties, such as the size, charge, density, and mass of spectroscopic features, are analyzed and associated with the protein corona [37].

The common applicability of DLS or TEM to the characterization of nanomaterials can also be extended to characterize the NPPC. In the case of DLS, the measurement of the hydrodynamic diameter of nanoparticles before and after the protein corona formation provides an estimation of the corona thickness [23]. Nevertheless, DLS estimation must consider the possibility of other potential factors, such as nanoparticle aggregation, affecting the analysis. TEM can also be used to determine the protein corona formation and size by assessing nanoparticles size before and after the formation of a protein corona. One of TEM limitations is the possibility of sample handling and preparation resulting in protein corona destabilization and degradation. Hence, cryo-electron microscopy (cryo-TEM) is preferred as it preserves the original state of the protein corona, even making it possible to discriminate between the soft and hard corona [40].

The study of nanoparticle–protein interactions can also be addressed with separation techniques such as size exclusion chromatography (SEC) or asymmetric flow field-flow fractionation (AF4). Nanoparticles are too large to be retained in the SEC phase pores, whereas proteins are not, and their elution time will depend on their molecular weight. Then, if proteins are associated with the nanoparticles, their retention time will be decreased, depending on the duration of the interaction [6]. This way, SEC can provide information on the proteins associated with the nanoparticle, which can also be separated from the non-associated proteins, as well as their association times. Alternatively, zeta-potential determination before and after the formation of the protein corona can also provide information on the thickness and effect of the protein corona on the nanoparticle surface charge. In this sense, Rampado et al. observed that when nanoparticles are incubated with proteins, the surface charge value tends toward a value of a few mV below zero, independent from the nanoparticles’ original charge [42].

## 3. The Need for Improved Methodologies in Nanoparticle–Protein Corona Analysis

### 3.1. Lack of Standardization in Nanoparticles Synthesis, Production and Characterization

The need to improve or dispose of more accurate and robust NPPC production and standard nanoparticle characterization methods is clear, as they would significantly improve reliability, reproducibility, and transparency in biomedicine [36]. The use of inadequately characterized starting nanoparticles can lead to errors in the subsequent evaluation of the NPPC biological behavior. Accurate characterization of nanoparticle size distribution is thus essential to avoid the formation of protein coronas with different composition due to the initial presence of a wide range of nanoparticle size populations in high polydisperse samples, or to control potential protein contaminations and the formation of aggregates. The indirect strategies often used to characterize NPPC imply a great number of steps and processes that lead to higher variability and potential errors due to nanoparticle alterations or losses. To minimize this risk, it is important to compare the size distribution of nanoparticles before and after the formation of the protein corona and consider how the size of the nanomaterial changes on interaction with a biological system.

The current methods to characterize nanoparticles, including DLS, DCS, or TEM, are highly dependent on instrumental conditions and sample preparation and are seldom validated, risking the compromise of the results by uncontrolled processes [43]. In recent years, there have been efforts to standardize the characterization of nanomaterials to reduce this lack of reproducibility and improve the robustness and accuracy of the methodologies. In the pursuit of this standardization, a group of leading researchers in the analytical nanotechnology field proposed MIRIBEL, a standardized reporting system to improve the quality of nanomaterial research and the way it is reported [44]. MIRIBEL suggests including high-quality, reproducible synthesis steps and considering factors such as size and shape, which can affect organ distribution and the adsorption of biomolecules onto nanomaterials. However, implementing MIRIBEL may be challenging for early-career scientists who lack critical resources and long-term experience in using various characterization techniques. The lack of standard methods hinders the comparison of different materials and the evaluation of the applicability of new design alternatives. Furthermore, there is a growing concern over the increasing nanomedicine products commercially available given the current lack of such control and standard nanoparticle characterization protocols [32]. 

### 3.2. Irreproducibility and Lack of Standardization in LC-MS Protein Analysis

The characterization of protein corona variable composition as a function of nanoparticle physicochemical properties (size, surface charge, morphology, etc.) cannot be assessed without reproducible and upright LC-MS analyses and datasets. For instance, researchers examined the interactions between proteins and nanoparticles and the effect of small changes in surface chemistry on these interactions using SDS-PAGE and LC-MS for protein separation and quantification, respectively [25]. The obtained results demonstrated that, for most of the proteins studied, their relative quantities were lower when nanoparticles were present. However, it should be noted that the identification of proteins by mass spectrometry was only performed once, which may exaggerate the differences between particle types. Thus, despite the central role of LC-MS in NPPC studies, the biological and technical variations can significantly compromise the reproducibility of analyses.

Validity of proteomics results depend on the robustness and reproducibility of preparation and characterization methods and can be compromised by biological and technical variations. Proteomics results are also dependent on the variability of identified proteins, which critically depends on the LC-MS instrumentation and data analysis approach [36]. Protein corona impurities (dependent on nanoparticle size) can be a significant source of contamination that may cause errors in proteomics analysis [40]. Unfortunately, the LC-MS technique suffers from variability in the proteomics datasets, which depend not only on the instrumentation, but also on the core facility [18,36]. This variability may lead to misinterpretation of NPPC biological, biomedical, therapeutical, or toxicological effects. Accordingly, protein quantification remains a challenge due to inherent limitations in LC-MS, the complex composition of the protein corona, and the lack of standard approaches. Different labs have access to different instrumentation, equipment, and software, which can affect data processing workflows and relative protein quantification. In this regard, the recent study of Ashkarran et al. is particularly relevant [18]. They carried out an interlaboratory study to determine the influence of different LC-MS/MS workflows in the analysis of identical aliquots of NPPCs by sending them to different proteomics facilities across the USA. Interestingly, the results provided by different laboratories showed significant differences in terms of identified and quantified species. In this regard, it must be remarked that mass spectrometry is inherently non-quantitative, hence protein quantification (necessary in protein corona kinetics studies) is more challenging than identification [45]. In fact, whereas quantification of target proteins is usually carried out using stable isotope-labeled protein analogous as standards, large-scale quantifications (as is the case when characterizing protein corona) are based on label-free approaches and are mostly relative quantitative approaches [41]. Ashkarran et al. found that, instead of proteomics methodology, parameters such as sample preparation, instrumental settings, and raw data processing are more relevant in the variability of analytical results. Another finding was the lesser biased in proteomics results when analyzing tissue or cell extracts in comparison to plasma fluids, given their lower protein dynamism, hence the less challenging nature of the analysis [46]. The high dynamism of protein concentration levels implies an added difficulty in proteomics analysis given that major proteins such as BSA, which accounts for more than half of the protein content in plasma [47], hinder the detection and accurate determination of minor proteins. Moreover, strategies for depleting abundant proteins from plasma are often unsuitable for use when analyzing a large number of samples due to high cost, difficult handling, and carry-over concerns. This type of study clearly demonstrates the need for standardization protocols and methods in MS protein analysis, especially at the quantitative level, that enable the comparison of data results across proteomics platforms and facilities. 

## 4. The Potential of ICP-MS in the Study of the Nanoparticle–Protein Corona Complex

### 4.1. Characterization of Nanoparticle Composition and Functionalization by ICP-MS

**Figure 4:**
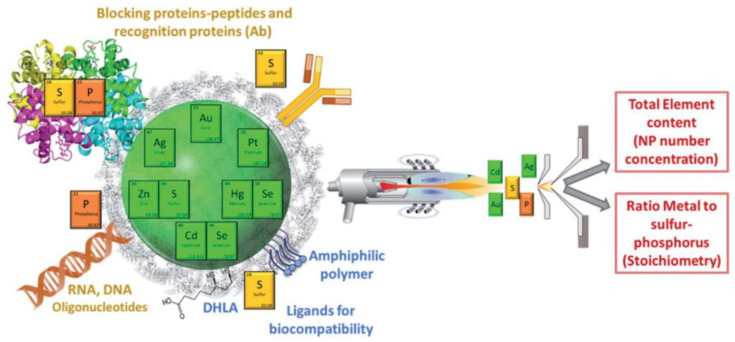
Use of ICP-MS for the characterization of nanoparticles. Reproduced from ref [27] with permission from the Royal Society of Chemistry.

Inductively coupled plasma mass spectrometry (ICP-MS) is a powerful elemental spectrometry technique that consists of the atomization and ionization of the sample in a high-energy argon plasma, which enables one to carry out multi-elemental and multi-isotope analysis. Th einorganic nanoparticles commonly used in bioanalytical applications are typically metal-based structures synthesized from metal-salt precursors. Thus, their metal atoms can be detected with ICP-MS to determine the multi-elemental content and absolute quantitative information on nanoparticle mass and number concentrations, as well as core/ligand ratios and elemental stoichiometries within the nanoparticle (Figure 4). Most ICP-MS approaches in protein corona analysis focus on nanoparticle metal quantification by elemental total analysis after sample digestion to dissolve the nanoparticles and remove matrix interfering compounds [48]. Such sample digestion is also necessary to prevent transport and nebulization issues in larger nanoparticles that would otherwise lead to biased quantitative results. The use of micro-flow total consumption nebulizers is a viable alternative to dispose of these effects [49,50]. Matrix interferences and contamination problems from inorganic metal target ions present in the sample entail prior sample purification approaches such as ultracentrifugation [51], or the application of an external magnetic field when working with magnetic nanoparticles [52].

The use of separation techniques coupled to ICP-MS opens the door to the analysis of nanoparticles in polydisperse samples. This is the case with size exclusion liquid chromatography, which has been used to determine the size and shape of metallic nanoparticles and quantum dots [53,54]. Asymmetric flow field-flow fractionation (AF4) coupled with ICP-MS is another nanoparticle fractionation technique used to avoid nanoparticle degradation or agglomeration of the nanoparticles that sometimes occurs with SEC during separation. With this method, nanoparticles are separated based on their hydrodynamic size, so that different nanoparticles can be separated and identified with AF4-ICP-MS with high recoveries (>81%) [55]. Furthermore, this approach can be used to characterize and quantify bioconjugates stoichiometries and populations [29]. It must be remarked that nanoparticle characterization with ICP-MS requires the nanoparticle to have an ICP-MS detectable element in its core. In the case of metallic nanoparticles, that element is the metal forming the core. In the case of non-metallic nanoparticles, ICP-MS detection can be performed if dopped with a detectable element, as is the case, for instance, of dopped carbon nanoparticles [56]. Protein formation dynamics in these cases can then be approached through the determination of the elemental ratio between the sulfur from the proteins, and the detectable element from the nanoparticle core [57]. It must be considered that the nanoparticles may contain sulfur or be functionalized with sulfur-containing ligands to provide stability and solubility to the nanoparticle. Sulfur/metal determination can also be used to determine stoichiometries ligand/nanoparticle, providing information of surface functionalization and ligand density. Nevertheless, in those cases, protein incorporation would be determined from the variation (increase) in the S/metal ratio respect to the initial ratio (corresponding to the surface ligands) [58].

Lastly, it is worth noting the increase in the use of the single particle-ICP-MS (spICP-MS) technique [59,60]. spICP-MS is based on the introduction of a very diluted sample into ICP-MS with a fast mass scanning speed configuration. Nanoparticle concentration and size can be obtained through signal intensity and pulse frequency, respectively. This technique allows elemental analysis of all types of matrices, because it provides the data needed to determine the size, size distribution, and particle number concentrations of nanoparticles with little time spent measuring the samples in suspension [61,62]. To translate ion intensity to ion mass, and assure the reproducibility and validity and establish metrological traceability of the methodology, adequate calibration approaches are required [60,63]. The most common calibration approaches are the use of well-characterized reference materials of nanoparticles with the same nature as the analyte, or the use of standard element solutions of the ICP-MS detectable element present in the analyte. Ideally the use of a nanoparticle reference material would provide the most straightforward calibration, given it is of known geometry and size and has the same composition as the nanoparticle analyte [63]. However, due to the scarcity of commercially available well-characterized reference materials, elemental standards solutions are often used for calibration in spICP-MS [60,64,65]. Their use for calibration, however, requires the determination of the nanoparticles transport efficiency, using strategies such as the determination of particle frequency or particle size methods using nanoparticles standards [66], methods based on the determination of the solvent transport efficiency such as dynamic mass flow (DMF) [64], or the use of low-volume, high-efficiency sample introduction systems such as total consumption nebulizers (TCN) [67].

It has already been discussed that determination of the protein corona thickness through the comparison of the nanoparticles and the NPPC size provides valuable information for studying the adsorption and interactions of the proteins with the nanoparticle. However, nanoparticle size determination with spICP-MS is approached through the measurement of the metal (or another detectable element) present in the nanoparticle core. In fact, size determination is based on the intensity of the detected events, and it is independent, as commented, of the nanoparticle environment. That is, for a certain nanoparticle, spICP-MS analysis would provide the core size exclusively and not the hydrodynamic size (ligands, protein corona, etc.). spICP-MS is moreover limited in terms of nanoparticle size, as it cannot determine sizes below 10 nm. [61]. Alternatively, strategies based on the combination of Taylor dispersion analysis (TAD) and ICP-MS have been proposed for the size determination of <10 nm nanoparticles [68]. In TDA, nanoparticle size is calculated through the determination of the diffusion coefficient, and the use of ICP-MS provides a significant enhancement in sensitivity compared to traditional approaches such as DLS. Because this approach determines the nanostructure size in the base to the gaussian fit of the peak obtained in TDA analysis, which is related to the hydrodynamic radius, it can differentiate between nanoparticles with and without the protein corona. In fact, the technique has been applied to the study of NPPC, proving useful to determine the nanoparticle size and the protein corona thickness, though it is still limited in terms of discriminating whether the increase in size is due to aggregation or the formation of a larger corona [69]. This approach is also restricted to studies with high protein concentration, and to small size complexes (<70 nm) for good accuracy.

### 4.2. Characterization of NPPC Protein Components by ICP-MS

ICP-MS sensitivity and specificity to detect trace levels of metals and other elements in biological samples (e.g., P or S) makes it useful for measuring the concentration of the proteins present in NPPC and so study its dynamic changes over time, making it a valuable tool for understanding the interactions between nanoparticles and proteins. In contrast to common molecular MS approaches for protein quantification (i.e., electrospray), ICP-MS ionization can be made species-independent. Once nebulization issues are under control [50], ICP-MS response becomes directly proportional to the mass concentration of the detected element, and independent of the protein structure, molecular weight, or charge state, requiring no specific protein standards for absolute quantification. Protein quantification analysis can be carried out through any ICP-detectable atom related to the protein (except C, H, O, and N). Once the element/protein molar ratio is known, the elemental signal of the target compound can be expressed as protein concentration. In this regard, sulfur detection can be considered as a generic ICP-MS approach for protein quantification, because S is present in cysteine (Cys) and methionine (Met) amino acid residues, and hence in most proteins. Therefore, when the number of these components (Met and Cys) is established, from the amino acid sequence, absolute protein quantification can be carried out by correlating with the sulfur quantified with ICP-MS [70]. This information can be obtained by parallel analysis with molecular sources such as ESI-MS [71,72,73]. It is worth remarking that the ICP-MS detectable element, however, can also be added externally, via, e.g., chemical reagents such as DOTA containing rare earth elements. In this regard, Liu et al. used ICP-MS to study the internalization of NPPC of Ru-containing NPs, used as drug nanocarriers, and the effect of their interaction with a cellular membrane glycopeptide, using an Eu-containing DOTA tagged to the peptide [74]. Through the detection of Ru and Eu with ICP-MS, they were able to study the effect of the corona on the internalization process and macrophage’s uptake.

It is important to consider that the separation dimension provided by chromatography is a prerequisite to detect and quantify each protein individually to be able to unequivocally correlate the elemental signal to a specific biomolecule. An example of this is the work carried out by Matczuk et al., who used capillary electrophoresis coupled to ICP-MS to determine the number of protein molecules bound to AuNPs of different sizes (5–50 nm). The use of CE permitted separation of the conjugate between AuNPs and albumin from the free AuNPs (in excess) and thus estimate the number of albumin molecules bound per AuNP, relating the peak areas obtained from the complex AuNP:albumin and the free unbound AuNPs [75]. Likewise, Fernández-Iglesias and Bettmer were able to estimate the total amount of proteins forming the hard corona of citrate-stabilized AuNPs, ranging from 10 to 60 nm, when incubated with human serum [76]. They calculated the total protein quantity from the sulfur concentration quantified with ICP-MS. To do so, they first confirmed with SEC-ICP-MS that the sulfur signal corresponded solely to protein species. Then, they carried out the washing and purification of the NPPC using centrifugation and sedimentation through sucrose, the latter being seemingly more efficient and requiring fewer washing steps. Finally, ICP-MS determination of the S/Au signal ratio was translated into number of proteins per nanoparticle, assuming an average number of 40 sulfur atoms per protein molecule.

Regardless of the large amount of potential information provided by ICP-MS, such as molar ratio NP/protein, absolute protein concentration, protein corona, or nanoparticle aggregation and/or size distribution, it is still necessary to combine this approach with previously described analytical methodologies for a better and complete characterization of the NPPC. For a better understanding of the different NP populations in protein corona studies, separation techniques should precede ICP analysis. Electrophoresis is commonly applied to determine binding kinetics and size exclusion, or hydrodynamic chromatography is suitable for species characterization [77,78]. Asymmetric flow-field flow fractionation can provide in a single run complementary information about the NP:protein ratio from free (i.e., non-surrounded by proteins) particles and different populations formed during bioconjugation [29]. In this regard, López-Sanz et al. used AF4-ICP-MS in combination with techniques such as UV-Vis spectroscopy, TEM, and ultracentrifugation (UC) to study and characterize AuNPs in cell culture media [79]. The combination of AF4 with ICP-MS provided valuable information on AuNPs transformations, such as oxidation processes in the cell media resulting in the formation of ionic Au, confirmed by ICP-MS after ultracentrifugation. The complementarity of ICP-MS with other techniques was further demonstrated in the characterization of protein corona formation. They observed longer AuNP retention time in AF4-ICP-MS, which was associated with an increase in their hydrodynamic size. The use of UV-Vis and TEM provided the necessary information to determine whether this increase in size was due to the formation of the protein corona or by aggregation of the nanoparticles, respectively.

## 5. Concluding Remarks and Prospects

Nowadays, it is unquestionable that the use of NPs in biological applications requires a precise knowledge of the NP–protein corona system (NPPC) that is formed when NPs enters biosystems. Consequently, NPs acquire a new biological identity through the formation of this protein corona, which affects their colloidal stability, biodistribution, interactions, toxicity, and clearance. The understanding of the fate of the NPPC in biological systems, the development of NPPCs with biomedical applications, or the understanding of the NPPC toxicological effect require accurate and reliable characterization of nanoparticle physicochemical properties, the protein corona composition, and the dynamics of the interaction between nanoparticles and the different proteins that constitute the corona. To this end, the combination of multiple analytical methodologies offering complementary information certainly seems to be necessary. Knowledge of nanoparticle protein corona composition commonly relies on approaches that lack standardization methods that guarantee or control the reproducibility and reliability of the results. In particular, important difficulties persist in the effective quantification of the different proteins from the corona. ICP-MS turns up as a complementary tool that could provide accuracy and robustness of quantitative results and workflows given its species-independent nature. In fact, thanks to the presence of ICP-detectable elements in widespread inorganic NPs (Au, Ag, Ti, Ce, Cd, Se, Zn, among other) and proteins (S), ICP-MS can provide valuable information, including NP elemental composition, size, concentration, and populations on the one hand, and absolute protein amounts on the other.

The combination of several state-of-the-art imaging techniques enable visualization of the individual biomolecules associated with the surfaces of NPs [40] and clearly demonstrate the great variability of the protein corona within the same sample. Notably, such highly valuable qualitative information provided by imaging techniques could be completed with the quantitative nature of the ICP-MS signal. However, ICP-MS itself cannot provide a comprehensive picture of the diverse NPPC populations present. It requires separation techniques that are able to first isolate free NPs from NPPS and then to assess the different protein: NP populations present in the sample. We anticipate that analytical platforms comprising adequate separation techniques (e.g., CE, SEC, AF4, HPLC) coupled on-line with ICP tandem MS detection (well suited for the sensitive and free-of-interference analysis of S) could be well suited to face the remarkable analytical challenge. We have also to keep on mind that the accurate and precise absolute protein quantification provided by ICP tandem MS falls short without information regarding the identity of the proteins involved in the NPPC. For that purpose, liquid chromatography coupled to mass spectroscopy (LC-MS/MS) remains the dominant methodology and is thus essential to achieve a detailed characterization of the protein corona.

In summary, we believe that ICP-MS could fill some gaps in NPPC characterization that are clearly identified and currently demanded and could be established as a useful and resourceful complementary tool in the current challenge of protein corona assessment.

## Data Availability

The data presented in this study are available on request from the corresponding author.
